# Diverse clinical features of symptomatic Meckel’s diverticulum: a multicenter study of 151 consecutive pediatric patients from the Western Balkans

**DOI:** 10.1007/s00383-025-06197-2

**Published:** 2025-10-04

**Authors:** Zlatan Zvizdic, Blagoje Grujic, Asmir Jonuzi, Edin Husaric, Vlatka Martinovic, Aleksandar Brkovic, Nikola Rakocevic, Amir Halilbasic, Valentina Lasic, Denis Pasalic, Emir Begagic, Semir Vranic

**Affiliations:** 1 Department of Pediatric Surgery, Clinical Center University of Sarajevo, Sarajevo, Bosnia and Herzegovina; 2 Institute for Mother and Child Health Care of Serbia “Dr. Vukan Cupic”, Belgrade, Serbia; 3 Department of Pediatric Surgery, Children’s Hospital, University Clinical Center Tuzla, Tuzla, Bosnia and Herzegovina; 4 Department of Pediatric Surgery, University Clinical Hospital Mostar, Mostar, Bosnia and Herzegovina; 5 Department of Pediatric Surgery, University Clinical Center of the Republika Srpska, Banja Luka, Bosnia and Herzegovina; 6 Department of Neurosurgery, Cantonal Hospital Zenica, Zenica, Bosnia and Herzegovina; 7 College of Medicine, QU Health, Qatar University, PO Box 2713, Doha, Qatar

**Keywords:** Meckel's diverticulum, Symptomatic, Surgery, Pediatric patients

## Abstract

**Purpose:**

Symptomatic Meckel’s diverticulum (MD) has various clinical presentations and can be easily misdiagnosed. This multicenter study examines the clinical characteristics, management, and outcomes of patients across five academic pediatric surgery centers in Bosnia & Herzegovina and Serbia.

**Methods:**

We retrospectively included all pediatric patients (< 18 years) who were surgically and histopathologically confirmed to have symptomatic MD between 2011 and 2020. Demographics, clinical and radiological features, surgical treatment approaches, histopathologic findings, and outcomes were collected and analyzed.

**Results:**

Among 151 patients (80.1% male), the median age was 6.7 years (IQR 1.5–10.8). Presentations included intestinal obstruction (38.4%), GI bleeding (37.8%), and peritonitis (23.8%); 63.6% had multiple symptoms. A technetium-99 m scan was positive in 80.7% of bleeding cases. Laparotomy was performed in 72.2%, laparoscopy in 23.2%, and conversion in 4.6%. Partial small bowel resection was required in 80.8%, versus diverticulectomy in 19.2% (p < 0.001). Ectopic mucosa was found in 55.6% (gastric 48.3%, pancreatic 2.6%, both 4.6%; p = 0.05), significantly more common in males (p < 0.001). Postoperative complications occurred in 3.2%, with no mortality.

**Conclusions:**

Symptomatic MD displays highly variable clinical presentations. It is often underdiagnosed preoperatively, particularly without GI bleeding, emphasizing the need for high clinical suspicion and tailored surgical approaches.

## Introduction

Meckel’s diverticulum (MD) is the most common congenital gastrointestinal (GI) abnormality, affecting 1.4–2% of the general population, as documented by large-scale autopsy and surgical studies [1]. MD arises when the omphalomesenteric duct (OMD) fails to involute fully or partially. Anatomically, MD is a true diverticulum, having all the layers of the intestinal wall, and most commonly arises from the antimesenteric border of the ileum, proximal to the ileocecal valve. The distance to the ileocecal valve varies depending on the patient's age, but it is most often within 60 cm proximally to the ileocecal valve [2].

It is usually asymptomatic with a ~ 5% lifetime complication risk, which decreases with age [3]. Symptomatic MD is virtually synonymous with a complication. Most MDs become symptomatic within the first two years of life and are a rare pathology in adult life. Despite being reported to affect both sexes equally [4], complications related to MD tend to affect males more frequently than females [4, 5]. When symptomatic, MD may present with intermittent, crampy abdominal pain, painless gastrointestinal (GI) bleeding, and bowel obstruction or diverticulitis with or without intestinal perforation [6]. From a clinical standpoint, making an accurate preoperative diagnosis in an acute setting is difficult due to the lack of a specific clinical presentation. However, any delay in diagnosis and treatment could have serious, life-threatening consequences.

Several studies have previously analyzed features of complicated MD, but without consistent results in the pediatric population [5–9]. There is a lack of detailed clinical information on symptomatic MD in South-eastern Europe, particularly in Bosnia and Herzegovina and Serbia, as only single case reports have been published [10]. Using a multi-institutional database, we designed the current study to examine clinical characteristics, treatment patterns, and outcomes in a population-based cohort of children and adolescents < 18 years with symptomatic MD in five academic hospitals in Bosnia and Herzegovina (n = 4) and Serbia (n = 1).

Based on a multicenter regional cohort, we hypothesized that symptomatic MD is frequently underdiagnosed preoperatively, particularly in cases without lower GI bleeding; that the type of complications, distribution of ectopic mucosa, and surgical approach vary by patient age and sex; and that partial small bowel resection is more commonly required than simple diverticulectomy in symptomatic cases, reflecting the anatomical location of ectopic tissue and the nature of the associated complications.

## Materials and methods

Patients < 18 years presenting with symptomatic MD between January 2011 and December 2020 at five academic hospitals in Bosnia and Herzegovina and Serbia were enrolled in the study. Participating academic hospitals included the Clinical Center University of Sarajevo, the University Clinical Center of Tuzla, the University Hospital Center Mostar, the University Clinical Center of the Republic of Srpska Banja Luka, and the Institute for Mother and Child Health Care of Serbia “Dr. Vukan Cupic”, Belgrade, Serbia.

Patients were identified through a review of medical records using the International Classification of Diseases, Ninth Revision, Clinical Modification (ICD-9-CM) code 751.0, which encompasses MD or remnants of the vitelline duct. Data regarding sex, age at presentation, clinical symptoms, preoperative diagnostic techniques, surgical treatment strategies, and histopathologic findings were gathered and analyzed. A 99mTc-pertechnetate scan, ultrasound, a computed tomography (CT) abdominal scan, surgical exploration, and histopathologic examination were utilized for diagnosing symptomatic MD. The presence of ectopic tissue was assessed in the resected diverticular specimen. Follow-up durations ranged from three to 12 years.

A minimum sample size of 109 patients was required for our research to have a 95% confidence interval (CI) and a 5% margin of error.

Based on the guidelines of the Eunice Kennedy Shriver National Institute of Child Health and Human Development in the United States, the patients were classified into five age groups: infancy (> 28 days to < 1 year), toddlerhood (1–2 years), early childhood (3–5 years), middle childhood (6–11 years), and early adolescence (12–18 years) [11].

The inclusion criteria were a surgically and histopathologically confirmed diagnosis of symptomatic MD and an age below 18 at the time of admission. The exclusion criteria were patients over the age of 18, incidental asymptomatic MD, incomplete or insufficient medical records, and surgery undertaken for unrelated abdominal pathology without suspicion of MD.

All patient medical records were de-identified and anonymized. Ethical approval was obtained from the Ethical Committee of the Clinical Center University of Sarajevo (ref. 0901–2-150/17), with informed consent waived due to the retrospective nature of the study. We adhered to STROBE reporting guidelines for observational studies.

**Fig. 1:**
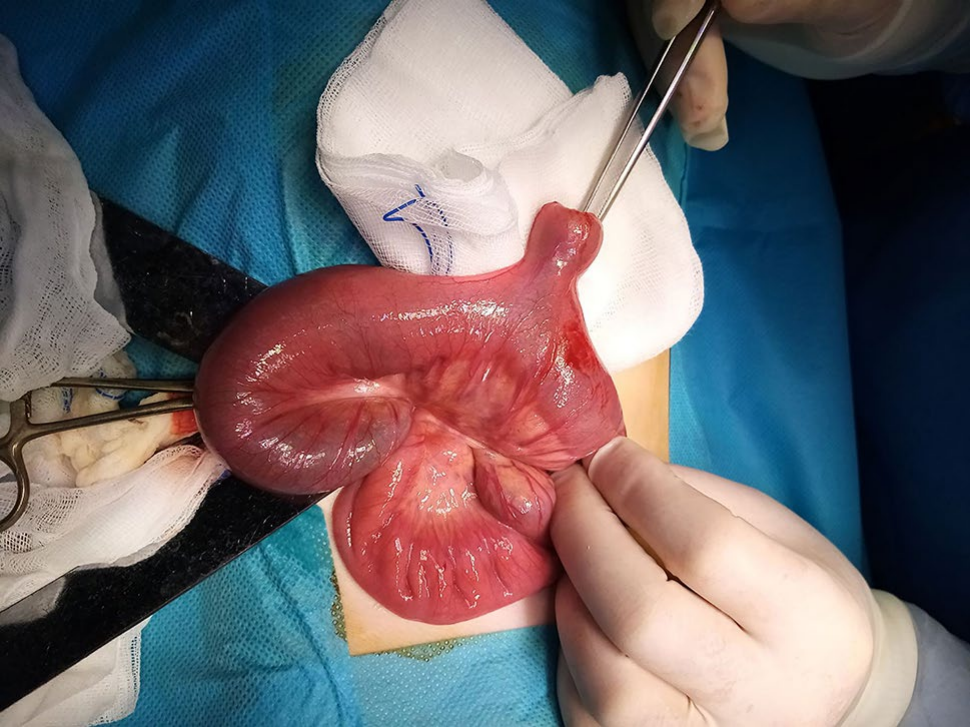
Perioperative view of Meckel’s diverticulum in a 4-year-old male patient with diverticular hemorrhage.

### Statistical analysis

Categorical variables were presented as numbers (percentage in total number), and Pearson's Chi-squared or Fisher’s Exact Tests were used for analysis. Continuous variables that did not follow a normal distribution or had outliers were presented as median (interquartile range, IQR), and the Mann–Whitney U test was used for analysis. All statistical analyses were performed in the Statistical Package for the Social Sciences (SPSS), IBM Version 27 (UNICOM Systems, Inc.). Statistical significance was defined as p < 0.05 .

**Fig. 2:**
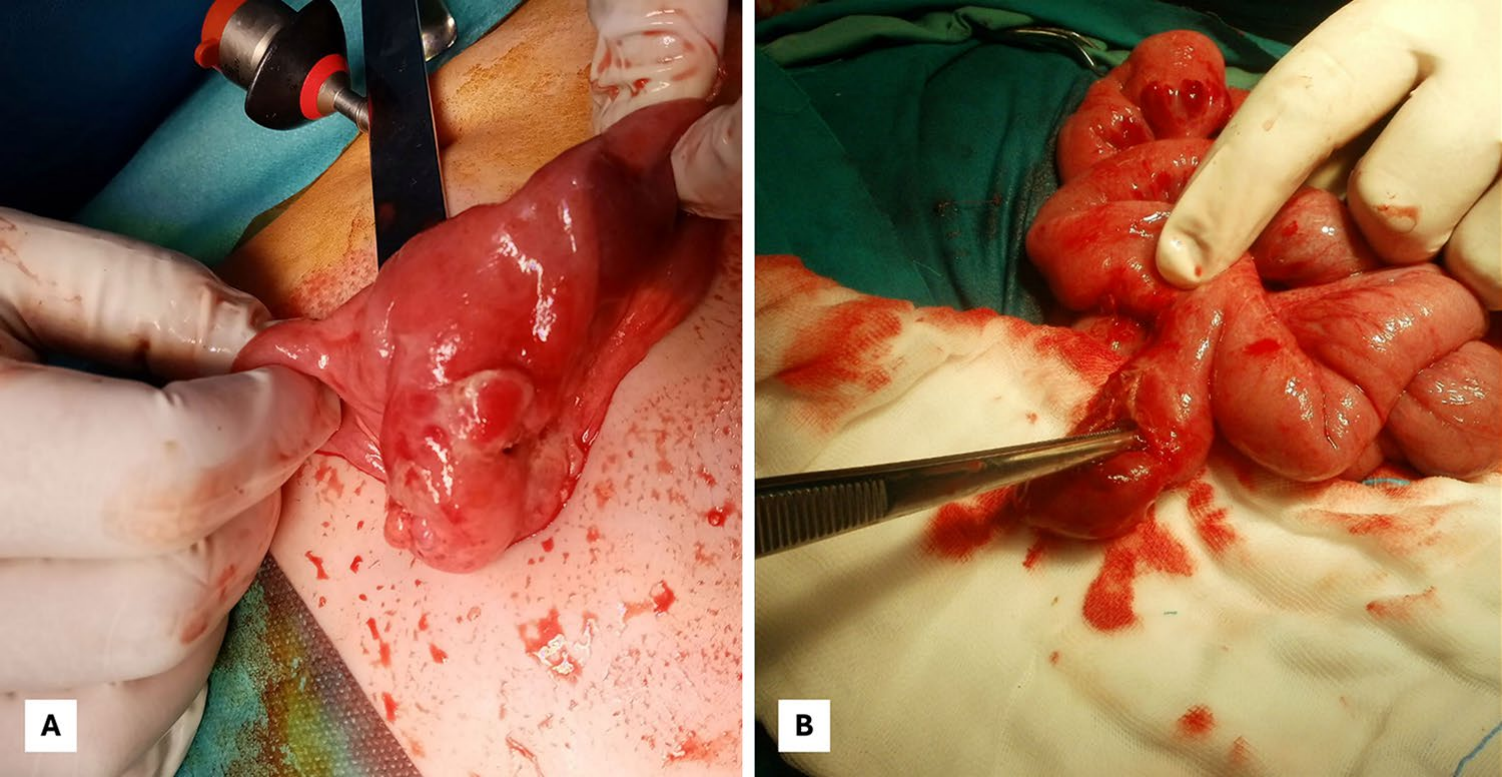
**A-B** Perioperative view of the perforated Meckel’s diverticulum exteriorized through the umbilical incision in an 8-year-old male patient (**A**); Perforated Meckel’s diverticulum in a 6-year-old male patient (**B**)

## Results

This multicenter retrospective analysis included 151 pediatric patients diagnosed with symptomatic MD. The cohort demonstrated a marked male predominance (80.1%), with a male-to-female ratio of approximately 4:1. The median age at presentation was 6.7 years (IQR, 1.5–10.8), with the highest incidence observed in middle childhood (41.7%) and early adolescence (20.5%).

The most frequent clinical presentation involved nonspecific abdominal pain, with or without nausea and vomiting, while gastrointestinal (GI) bleeding was documented in 37.8% of patients.Fig.1 Importantly, 63.6% of children were presented with more than one symptom, underscoring the heterogeneous clinical spectrum of symptomatic MD.

Complications were common, with intestinal obstruction identified in 38.4% and peritonitis in 23.8% of patients.Fig.2 In total, 62.3% were presented with features of an acute abdomen. A subset of patients in our cohort presented clinical features suggestive of appendicitis (119/151, 78.8%) and underwent emergency surgery, with a preoperative diagnosis of suspected appendicitis. During the surgical exploration, a symptomatic MD was identified intraoperatively. Notably, among non-bleeding cases, the correct preoperative diagnosis of MD was achieved in only 3.2%, reflecting significant diagnostic limitations.

Imaging modalities were largely uninformative: abdominal ultrasound failed to detect MD in any patient, and CT identified MD in only 5 (11.9%) of the 42 patients scanned. In contrast, technetium-99 m pertechnetate scans were positive in 46 of 57 (80.7%) patients presenting with GI bleeding (*p* < 0.001), underscoring their diagnostic utility in cases with bleeding manifestations.

Surgical management was dominated by open laparotomy (109/151, 72.2%), while laparoscopy was utilized in 23.2% (35/151) of cases, with a conversion to open surgery in seven out of 35 patients. The preferred operative technique was segmental small bowel resection with primary anastomosis (80.8%, 122/151), substantially more frequent than simple diverticulectomy (19.2%, 29/151), reflecting the complexity and extent of pathological involvement.

Among cases of intestinal obstruction (n = 58), intussusception was the leading cause (72.4%), with ileo-ileal invagination accounting for 64.3% of these. In 54.7% cases, surgical intervention followed at least one failed attempt at hydrostatic reduction. Among the 36 peritonitis cases, 47.2% were due to perforated MD, and 52.8% due to Meckel’s diverticulitis.

Histopathological analysis revealed ectopic tissue in 55.6% of cases, most commonly ectopic gastric mucosa (48.3%), followed by combined gastric and pancreatic tissue (4.6%), and ectopic pancreas alone (2.6%). The presence of ectopic mucosa was significantly more common in males (p < 0.001) (Table 1).

**Table 1:** Demographic and clinical characteristics of pediatric patients with symptomatic Meckel’s diverticulum (MD)

Characteristic	Overall N = 151 (100%)	Intestinal obstruction N = 58 (38.4%)	Gastrointestinal bleeding N = 57 (37.8%)	Peritonitis N = 36 (23.8%)			p-value
*Sex*							** < 0.001**
Male	121 (80.1)	47 (81.0)	50 (87.7)	24 (66.7)			
Female	30 (19.9)	11 (19.0)	7 (12.3)	12 (33.3)			
**Age, years†**	6.7 (IQR 10.8–1.5)	7.15 (IQR 10.8–1.6)	6.3 (IQR 10.8–1.4)	10.8 (IQR 12.7–7.4)			
Male-to-female ratio	4:1	4.3:1	7.1.1	2.0:1			
Age, group*							
Infancy	25 (13.9)	9 (15.0)	12 (21.1)	4 (11.1)			
Toddlerhood	15 (9.9)	9 (15.0)	6 (10.5)	0 (0)			
Early childhood	17 (11.3)	8 (13.8)	9 (15.8)	0 (0)			
Middle childhood	63 (41.7)	22 (37.9)	19 (32.8)	22 (37.9)			
Early adolescence	31 (20.5)	10 (32.3)	11 (35.4)	10 (32.3)			
Male-to-female ratio	4:1						
Infancy	5.3:1	9:0	3.5:1	1:1			
Toddlerhood	14:1	8:1	7:0	-			
Early childhood	4.7:1	3:1	8:1	-			
Middle childhood	2.5:1	2.1:1	5.3:1	2.1:1			
Early adolescence	5.2:1	9:1	10:1	2.3:1			
*Presenting signs and symptoms*							
Nonspecific abdominal pain with/without nausea or vomiting GI bleeding Fever	89 (58.9) 79 (52.3) 40 (26.5)	52 (89.7) 20 (34.5) 16 (27.6)	6 (10.5) 57 (100) 5 (8.8)		31 (88.9) 2 (5.6) 19 (52.8		
*Imaging findings (any MD detected)*							
Ultrasound	0	0	0	0			
Computerized Tomography	5/42 (11.9)	1/14 (7.1)	-	4/28 (14.3)			
Technetium-99 m pertechnetate scan	46/57 (80.7)	-	46 (80.7)	-			** < 0.001**
*Surgical approach*							
Laparotomy	109 (72.2)	51 (46.8)	54 (49.5)	4 (3.7)			** < 0.001**
Laparoscopy	42 (27.8)	7 (16.7)	3 (7.1)	32 (76.2)			
Conversion to laparotomy	7 (16.7)	2 (28.6)	1 (14.3)	4 (57.1)			**0.003**
*Surgery procedure*							** < 0.001**
Diverticulectomy	29 (19.2)	9 (15.5)	6 (10.5)	14 (38.9)			
Partial resection of the small intestine	122 (80.8)	49 (84.5)	51 (89.5)	22 (61.1)			
*Histopathologic findings/mucosa type*							
Normal mucosa	67 (44.4)	47 (81.0)	-	20 (55.6)			0.05
Ectopic mucosa	84 (55.6)	11 (19.0)	57 (100)	16 (44.4)			
Gastric	73 (48.3)	6 (10.3)	52 (91.2)	15 (41.7)			
Pancreatic	4 (2.6)	3 (5.2)	-	1 (2.8)			
Gastric and pancreatic	7 (4.6)	2 (3.4)	5 (8.8)	-			
*Ectopic mucosa*							** < 0.001**
Male	74 (88.1)	7 (12.1)	55 (96.5)	12 (33.3)			
Female	10 (11.9)	4 (6.9)	2 (3.5)	4 (11.1)			
Male-to-female ratio	7.4:1	1.8:1	27.5:1	3:1			

Data presented as n (%) and analyzed using Pearson's Chi-squared test or Fisher’s Exact Test or †median (interquartile range, IQR) and analyzed using the Mann–Whitney U test

^*^Neonates = less than 28 days; Infancy = 28 days to 1 year; Toddlerhood = 1–2 years; Early childhood = 3–5 years; Middle childhood = 6–11 years; Early adolescence = 12–18 years

Postoperative complications were observed in 5.3% of patients, primarily including postoperative ileus (n = 5) and wound infections (n = 3). One case of ventral hernia was noted as a secondary complication of wound infection in the form of ventral hernia. Importantly, all patients recovered, and no postoperative mortality was reported during a follow-up period ranging from 3 to 12 years.

## Discussion

Our multicenter cohort of 151 pediatric patients from Bosnia and Herzegovina and Serbia demonstrated that GI bleeding, intestinal obstruction, and peritonitis are the most frequent presentations of symptomatic MD. Notably, preoperative diagnosis was accurate in only 3.2% of non-bleeding cases, reaffirming the hypothesis that symptomatic MD is frequently underdiagnosed outside of bleeding presentations.

Although MD is the most common congenital GI abnormality, with a reported prevalence of ~ 1–2% in the general population [1, 12], symptomatic MD occurs in only 4–6% of cases [13]. Similar to the higher prevalence of MD in male patients than in female patients, with a ratio of 1.5:1 to 4:1 [1, 12], the symptomatic presentation of MD is also more common in males than in females [4, 5]. The male-to-female ratio in neonates ranges from 6:1 to 9:1 [14], whereas in older symptomatic children, the male-to-female ratio is 3:1 to 5:1 (Table 2) [15, 16]. The male predominance (4:1) and higher occurrence of ectopic gastric mucosa in our study align with previous reports [1, 4], [5, 15, 17], potentially influenced by regional dietary or genetic factors affecting gastric mucosa prevalence, and contribute to a higher rate of symptomatic cases (ulceration, bleeding, or perforation). Although the cause of this male predominance remains unclear, it may be attributed to the higher gastrin and acid levels in males, which affect the ectopic gastric mucosa and increase the risk of ulceration, bleeding, and obstruction [17]. A preoperative diagnosis of MD complications in an acute setting is challenging since the clinical manifestations of complicated MD might mimic any intra-abdominal emergency, such as intestinal obstruction, perforation with peritonitis, and abscess formation. This diversity of clinical presentation, coupled with the relative uncertainty of diagnostic testing, is the primary reason for the low preoperative symptomatic MD detection rate. Ludtke et al. and Bani-Hani et al. reported that, excluding cases presenting with bleeding, only 4% and 5.9% of their cases, respectively, received an accurate preoperative diagnosis [3, 18]. Likewise, among our symptomatic patients who presented with non-bleeding symptoms, only 3.2% received an accurate preoperative diagnosis of symptomatic MD. In our cohort, ~ 79% of patients presented with clinical features suggestive of appendicitis and underwent emergency surgery for presumed appendicitis. However, intraoperative findings revealed a symptomatic MD, underscoring the diagnostic challenge posed by MD when it mimics appendicitis. This aligns with previous studies that highlight the varied and often nonspecific presentation of MD in children, which can easily be mistaken for other abdominal emergencies, such as appendicitis. Chen et al. reported that MD can manifest in multiple ways, ranging from asymptomatic cases to severe complications like obstruction and perforation, often presenting in a manner indistinguishable from other abdominal pathologies, including appendicitis [9]. Their study also emphasized the need for careful diagnostic evaluation and heightened awareness of MD in pediatric patients with acute abdominal pain, even when no bleeding is present [9]. Reported complications of symptomatic MD include hemorrhage, perforation, inflammation, intestinal obstruction, stone formation, and hernia (Table 2) [7, 19]. It is well documented that symptomatic MD is most often presented in the youngest children in the form of painless or minimally painful GI bleeding [20, 21]. However, some studies reported intestinal obstruction as the most common symptom in younger pediatric populations (Table 2) [16, 22]. The incidence of complications in the form of bleeding and intestinal obstruction was almost equal in our study. Age-related data from the literature indicate that bleeding is most frequently observed in children < 2 years, while intestinal obstruction tends to occur more commonly in adults [23]. However, some research has reported the opposite [16]. Like other studies [6, 8, 24], ~ 80% of patients in our cohort were in the pre-adolescent age group, with the youngest patient being ~ six years old, and this age group was more commonly associated with lower GI bleeding.

**Table 2:** Comparison of symptomatic Meckel’s diverticulum in pediatric population: Our study vs. published literature

Study (country)	Patients (n)	Male: female ratio	Median/mean Age	Presentation (%)	Preop. diagnosis accuracy	Ectopic mucosa (%)	Type of surgery (%)	Mortality
Current study (Western Balkans 2025)	151	4:1	6.7 yrs (IQR 1.5–10.8)	Obstruction 38.4% Bleeding 37.8% Peritonitis 23.8%	3.2% in non-bleeding	55.6 (gastric 48.3)	Small bowel resection 80.8% Diverticulectomy 19.2%	0%
Park et al. 2005 (Mayo Clinic, USA) [16]	238 (58 children)	~ 3:1	All ages	Obstruction 40%, bleeding 31%, and obstruction 38% in adults	Not specified	59%	Not specified	0%
Tseng and Yang 2009 (Taiwan) [22]	45	~ 3:1	5.6 yrs (IQR 0.1–14.7)	Bleeding 46.7% Obstruction 26.7% Diverticulitis 26.7%	~ 3%	46.3%	Not specified	0%
Rattan et al. 2016 (India) [6]	65	4:1	3.2 yrs	Obstruction 86.1%, peritonitis 9.1% Bleeding 4.6%	5–6%	Not specified	Not specified	1.5%
Chen et al. 2018 (China) [9]	233	3.3:1	2.8 yrs	Bleeding 42.5, Obstruction 32.2% Peritonitis 24.0	~ 5%	66.1%	Resection 75% Diverticulectomy 25%	0.4%

The technetium-99 m pertechnetate imaging positivity rate was recorded in 80.7% of our patients, agreeing with the results of other studies [25–27]. However, since technetium-99 m pertechnetate imaging was performed only on patients with lower GI bleeding in emergency settings, we could not assess its sensitivity and specificity for the entire cohort.

The length of MD and the positioning of the ectopic gastric mucosa significantly influence surgical treatment [28]. According to the findings of Mukai et al., long diverticula (> 1.6 times the height-to-diameter ratio) have ectopic gastric mucosa only in the distal area [28]. In comparison, short diverticula (< 1.6 times the height-to-diameter ratio) have ectopic gastric mucosa in almost all areas [28]. Since we did not determine the precise height-to-diameter ratio of MD, the choice of surgical intervention (whether to perform a diverticulectomy or a partial resection of the small intestine) was based on the attending surgeon's preference and/or institutional protocols. Our decision to perform partial small bowel resection significantly more frequently than diverticulectomy was also related to the known fact that in symptomatic cases of MD, ectopic gastric mucosa is usually located in the basal part; therefore, diverticulectomy performed without removal of the accompanying segment of bowel may not be appropriate [29].

The role of laparoscopy in the diagnosis and treatment of bleeding MD in children merits further discussion. While technetium-99 m pertechnetate scanning remains the primary non-invasive diagnostic modality for detecting ectopic gastric mucosa, its accuracy can be limited by factors such as active bleeding or small lesion size [25–27]. In such cases, diagnostic laparoscopy serves as a valuable adjunct or alternative, offering both visualization and therapeutic resection of the diverticulum in a single procedure. Several studies have shown that laparoscopy is useful when preoperative imaging is inconclusive and clinical suspicion remains high [6, 24, 29]. For patients presenting with gastrointestinal bleeding of uncertain origin, laparoscopy allows for prompt localization and management of the bleeding source, particularly when combined with laparoscopic-assisted small bowel examination. Additionally, minimally invasive surgery offers pediatric patients benefits including shorter hospital stays, reduced postoperative pain, and faster return to normal activity [6, 7, 24]. Given these advantages, laparoscopy should be considered a first-line surgical approach in select cases of suspected bleeding caused by MD in children.

The findings of this multicenter study support all proposed hypotheses. First, symptomatic MD was frequently underdiagnosed preoperatively, particularly in patients without GI bleeding, due to its non-specific presentation and limited sensitivity of standard imaging modalities outside of technetium-99 m scans. Second, the type of complications, the presence and distribution of ectopic mucosa, and the choice of surgical intervention varied significantly by patient age and sex. Notably, male patients demonstrated a significantly higher incidence of ectopic gastric mucosa and symptomatic complications. Third, partial small bowel resection was more common than simple diverticulectomy in symptomatic cases, reflecting the anatomical distribution of ectopic tissue and the extent of pathological changes requiring broader resection. These findings underscore the importance of maintaining a high index of suspicion and tailoring surgical approaches to individual patient factors.

This study has several limitations. Firstly, it was a retrospective study with a relatively small sample size. Secondly, the information gathered was confined to what was present in the medical records. Finally, the generalizability of findings and comments on the sensitivity, specificity, or positive and negative predictive values is not feasible because the study design did not include a control group, and our results are presented solely as absolute numbers and percentages.

In conclusion, our multicenter study highlights that symptomatic Meckel’s diverticulum (MD) is often underdiagnosed preoperatively, particularly in patients without gastrointestinal bleeding. The study reinforces the male predominance of symptomatic MD and shows that the type of complications, distribution of ectopic gastric mucosa, and surgical approach vary significantly by age and sex. Partial small bowel resection was more frequently performed than diverticulectomy, reflecting the extent of pathology. These findings underscore the importance of early recognition and individualized surgical management for pediatric patients with symptomatic MD, and they support the need for heightened clinical suspicion, especially in non-bleeding cases. Further studies with larger sample sizes and prospective designs are warranted to validate these findings and explore optimal diagnostic and treatment strategies.

## Data Availability

No datasets were generated or analysed during the current study.
